# Postoperative Delirium and Cognitive Dysfunction after General and Regional Anesthesia: A Systematic Review and Meta-Analysis

**DOI:** 10.3390/jcm12103549

**Published:** 2023-05-18

**Authors:** Dmitriy Viderman, Fatima Nabidollayeva, Mina Aubakirova, Dinara Yessimova, Rafael Badenes, Yerkin Abdildin

**Affiliations:** 1 Department of Biomedical Sciences, Nazarbayev University School of Medicine (NUSOM), Kerei and Zhanibek Khandar Str. 5/1, Astana 010000, Kazakhstan; drviderman@gmail.com (D.V.); dinara.yessimova@nu.edu.kz (D.Y.); 2 School of Engineering and Digital Sciences, Nazarbayev University, 53 Kabanbay Batyr Ave., Astana 010000, Kazakhstan; 3 Department of Anaesthesiology and Intensive Care, Hospital Clínico Universitario de Valencia, University of Valencia, 46010 Valencia, Spain

**Keywords:** general anesthesia, regional anesthesia, post-operative delirium, post-operative cognitive dysfunction

## Abstract

**Background:** Perioperative disorders of neurocognitive function are a set of heterogeneous conditions, which include transient post-operative delirium (POD) and more prolonged post-operative cognitive dysfunction (POCD). Since the number of annually performed surgical procedures is growing, we should identify which type of anesthesia is safer for preserving neurocognitive function. The purpose of this study was to compare the effect of general anesthesia (GA) and regional anesthesia (RA) in patients undergoing surgical procedures under general anesthesia and regional anesthesia. **Material and methods:** We searched for randomized controlled studies, which studied post-operative cognitive outcomes after general and regional anesthesia in the adult patient population. **Results:** Thirteen articles with 3633 patients: the RA group consisted of 1823 patients, and the GA group of 1810 patients, who were selected for meta-analysis. The overall effect of the model shows no difference between these two groups in terms of risk for post-operative delirium. The result is insensitive to the exclusion of any study. There was no difference between RA and GA in terms of post-operative cognitive dysfunction. **Conclusions:** There was no statistically significant difference between GA and RA in the incidence of POD. There was no statistically significant difference in the incidence of POCD per-protocol analysis, psychomotor/attention tests (preoperative/baseline, post-operative), memory tests (postoperatively, follow up), mini-mental state examination score 24 h postoperatively, post-operative reaction time three months postoperatively, controlled oral word association test, and digit copying test. There were no differences in the incidence of POCD in general and regional anesthesia at one week postoperatively, three months postoperatively, or total events (one week or three months). The incidence of post-operative mortality also did not differ between two groups.

## 1. Introduction 

Perioperative disorders of neurocognitive function are a set of heterogeneous conditions, including post-operative cognitive dysfunction (POCD) and post-operative delirium (POD) [1]. POCD is one of the most common complications in the elderly patient population after surgery under general anesthesia [2,3,4]. POCD is characterized by a new cognitive impairment that occurs after a surgical procedure [2]. Its manifestations are subtle and manifold, depending on the affected cognitive function. The most commonly seen problems are memory impairment and impaired performance on intellectual tasks [2]. POCD involves several cognitive domains, such as attention, memory, and executive functions [5,6]. In turn, attention impairment in POCD combines disorder of the following independent networks: (1) alerting, (2) orienting, and (3) executive control, which is distinguished at the biochemical and cognitive levels [5,6,7,8]. The diagnosis requires both pre- and post-operative psychometric testing. Several previous studies reported long-term POCD in elderly patients.

It has been consistently speculated that the risk of POCD might be mitigated if surgical procedures are performed under local or regional anesthesia. However, previous studies did not find a significant difference when comparing general anesthesia and regional anesthesia using neuropsychological testing [5,9,10].

Hypothetical mechanisms of POCD include surgical trauma and neuroinflammation through disruption of the blood–brain barrier (BBB), leading to functional disruption of neural activity and POCD [1]. Each element of this hypothesis is controlled by a variety of inflammatory mediators. These events can persevere long-following surgery resulting in neurocognitive decline, especially in frail patients [1].

The second type of disorder of neurocognitive function is delirium. Delirium is an “organ failure of the brain”. Post-operative delirium (POD) is a frequent neuropsychiatric post-operative complication, predominantly in elderly patients [11]. The incidence of POD is reported to occur from 10% to 70% of patients depending on patient age, comorbidities, and type of surgery [12].

POD worsens short- and long-term outcomes, associated with high morbidity and mortality rates, high post-operative complication rates, prolonged intensive care unit (ICU) and hospital stay, loss of independence, long-term disability, increased hospitalization cost, and medication use [13]. POD is associated with an increased risk of persistent cognitive dysfunction and dementia [14]. Cognitive dysfunction is identified using a series of neuropsychiatric tests that offer a detailed assessment of higher cortical function rather than the usual neurological examination. It is difficult to determine the precise cause of POCD, for example, surgery-related or anesthesia-related, and these causes are currently almost inseparable.

Taking into account that the proportion of the elderly population and the number of surgical procedures in this population is growing rapidly, it is important to find the anesthetic method with the least negative effect on cognitive function. Since the percentage of the elderly population is expected to increase, the burden of this problem is expected to increase as well [11,12,13].

The purpose of this study was to compare cognitive outcomes in patients undergoing surgical procedures under general anesthesia (GA) and regional anesthesia (RA).

## 2. Material and Methods

### 2.1. Protocol

We followed the “Preferred Reporting Items for Systematic Reviews and Meta-Analyses (PRISMA)” [15]. The protocol was registered in PROSPERO (registration number: CRD42022306582). We searched for prospective studies which studied post-operative cognitive outcomes after general and regional anesthesia in the adult patient population.

**Figure 1:**
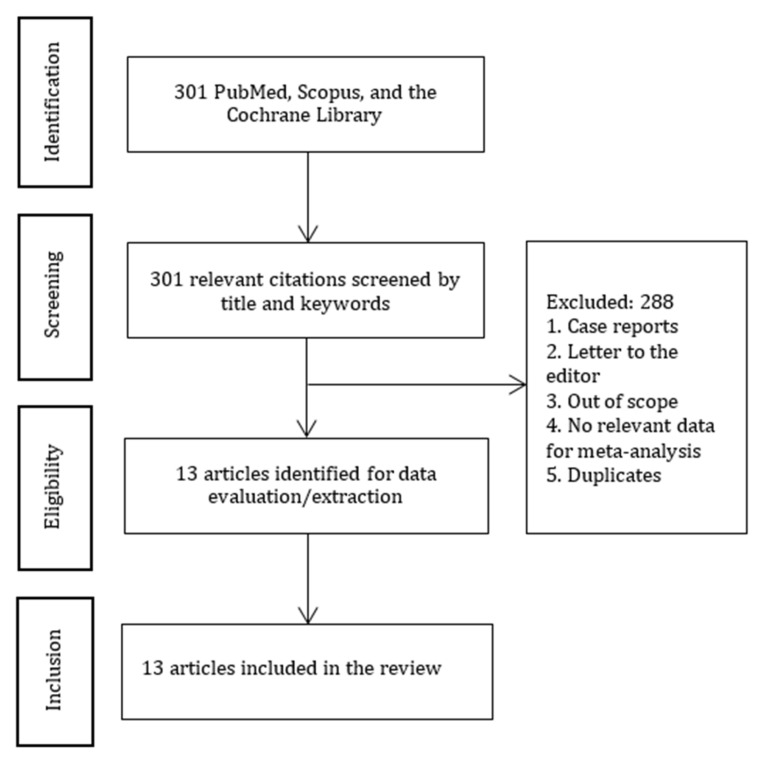
PRISMA diagram.

One of the authors searched for relevant articles in PubMed, Scopus, and the Cochrane Library published before April 2023 (Figure 1). All authors participated in data extraction.

The following search terms or their combination: “post-operative cognitive outcomes”, “post-operative cognitive dysfunction”, “post-operative delirium”, “general anesthesia”, “regional anesthesia”, “spinal anesthesia”, “epidural anesthesia”, “local anesthesia”, “femoral nerve block”, “sciatic nerve block”, “cervical plexus block”, “brachial plexus block”, “erector spinal plane block”, “transversus abdominis plane block”, and “paravertebral block” were used during the search.

### 2.2. Participants and Population

#### 2.2.1. Inclusion Criteria

This meta-analysis included randomized or partially randomized controlled trials of patients 18 years and older who were under general anesthesia (GA) or regional anesthesia (RA) after any type of surgery.

#### 2.2.2. Exclusion Criteria

Other types of studies (retrospective, case reports, case series, editorials, cadaver studies, and technical reports) and studies that did not adequately describe study methodology, evaluation, and/or reporting methods were excluded.

#### 2.2.3. Outcomes

By post-operative cognitive outcomes, we imply post-operative cognitive dysfunction or post-operative delirium. To assess post-operative cognitive outcomes, we used the following tests: the post-operative mini-mental state examination (MMSE) score 24 h postoperatively, psychomotor/attention (namely, a digit span, a digit symbol, a trail-making test A and trail-making test B) tests’ outcomes (preoperative/baseline and post-operative), memory tests’ outcomes (post-operative and follow up), and digit symbol test.

The primary aim of our meta-analysis was to analyze the influence of the type of anesthesia on post-operative delirium and post-operative cognitive dysfunction. The following variables and tests were analyzed: psychomotor/attention tests (preoperative/baseline as well as post-operative), memory tests (post-operative and follow-up), MMSE score 24 h postoperatively, post-operative reaction time three months postoperatively, controlled oral word association test, and digit copying test. The secondary outcome was death during three months.

### 2.3. Data Extraction and Statistical Methods

We extracted and entered data in the table. The following rubrics were included: authors, year of publication, country, reference, design, and goals of the study, age of participants, type of surgery, sample size, “American Society of Anesthesiologists (ASA) physical status”, and side effects. Data analysis was performed using the “Review Manager software (RevMan, version 5.4)”. Statistical heterogeneity was estimated using the I^2^ statistic.

### 2.4. Assessment of Methodological Quality

We assessed the methodological quality of the included studies using the Cochrane Risk of Bias Tool. For the assessment of the certainty of the evidence of primary outcomes, we used “the Grading of Recommendations, Assessment, Development, and Evaluation” (GRADE).

## 3. Results

In total, 301 articles matched our search criteria. Thirteen articles (Campbell 1993 [16]; Casati 2003 [17]; Ghoneim 1988 [18]; Haan 1991 [19]; Jones 1990 [20]; Li 2022 [21]; Neuman 2021 [22]; Rasmussen 2003 [10]; Silbert 2014 [23]; Tzimas 2018 [24]; Weber 2009 [25]; Williams-Russo 1995 [26]; Zhang 2019 [27]) with 3633 patients (RA group—1823 and GA group—1810) were selected for meta-analysis (Figure 1). The characteristics of the studies, the patients, the types of surgeries, and the anesthetic techniques are summarized in Supplementary File S1, “Characteristics of included studies”.

### 3.1. Incidence of Post-operative Delirium

**Figure 2:**
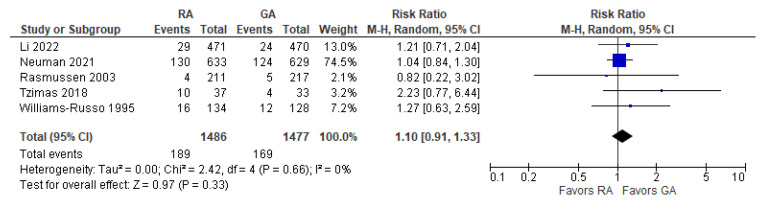
Incidence of post-operative delirium [10,21,22,24,26].

The incidence of post-operative delirium was reported in five [10,21,22,24,26] studies (Figure 2). The overall effect of the model shows no significant difference between RA and GA (risk ratio, RR, with 95% CI: 1.10 [0.91, 1.33], *p*-value = 0.33, I^2^ = 0). 

### 3.2. Incidence of POCD Per-Protocol Analysis

**Figure 3:**
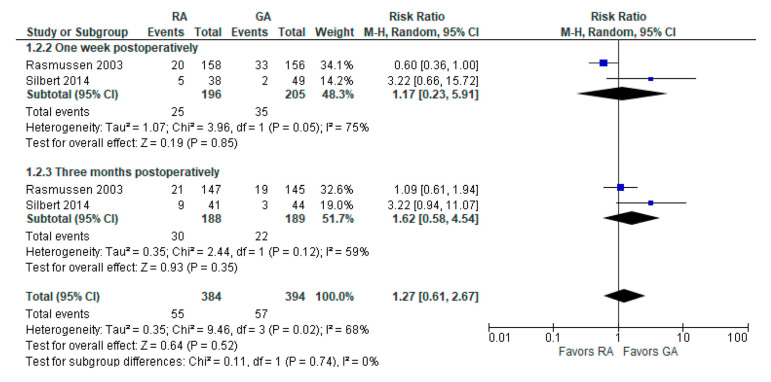
Incidence of POCD per-protocol analysis [10,23].

The incidence of POCD was reported in two [10,23] studies. The overall effect of the model (Figure 3) shows no risk difference between the RA group and the GA (RR with 95% CI: 1.27 [0.61, 2.67], *p*-value = 0.52, I^2^ = 68% (moderate)). The subgroup analysis for one week postoperatively and three months postoperatively shows no difference either. However, in one week postoperatively, the result is sensitive to the exclusion of Silbert 2014 [23]: the model tends to favor RA over GA.

### 3.3. Psychomotor/Attention Tests (Preoperatively/Baseline)

**Figure 4:**
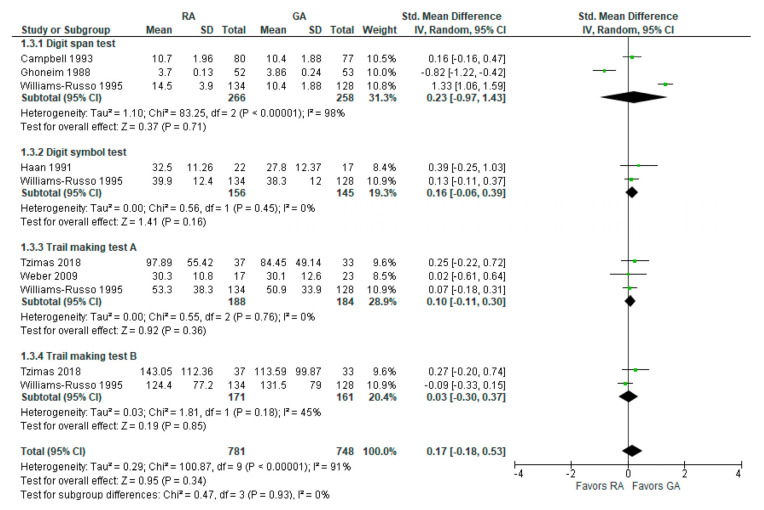
Psychomotor/attention tests (preoperative/baseline) [16,18,19,24,25,26].

The psychomotor/attention (preoperative/baseline) tests’ results were reported in six [16,18,19,24,25,26] studies (Figure 4). Weber et al., 2009 [25] reported (the German version) trail-making test results; we incorporated it as A test (A subgroup). The overall effect of the model does not show any difference between RA and GA (SMD with 95% CI is 0.17 [–0.18, 0.53]), and this result is insensitive to the exclusion of any study. In subgroup analysis, the model does not show any difference between RA and GA in all subgroups.

### 3.4. Psychomotor/Attention Tests (Postoperatively)

**Figure 5:**
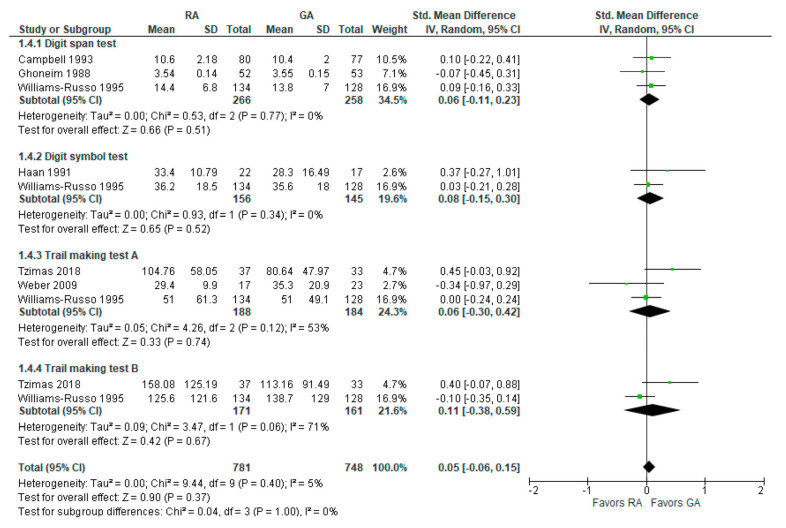
Psychomotor/attention tests (postoperatively) [16,18,19,24,25,26].

The psychomotor/attention (post-operative) tests’ results were reported in six [16,18,19,24,25,26] studies (Figure 5). For a digit span test, three studies [16,18,26] reported measured results in three different time intervals: two weeks, mean one to ten days, and one week postoperatively. In this subgroup analysis, the model does not show any difference between RA and GA. For a digit symbol test, the model based on two studies [19,26] does not show any difference between RA and GA. The data values were given as a mean of four days [19] and at one week postoperatively [26].

In the trail-making test A conducted by the studies [24,25,26], the model does not show any difference between RA and GA. Data values were reported as a mean of 30 days [24] and one week postoperatively [26]. In trail-making test B, the model does not show any difference between RA and GA. The overall effect of the model shows no difference between RA and GA (SMD with 95% CI is 0.05 [–0.06, 0.15]), and this result is insensitive to the exclusion of any study.

### 3.5. Visual Recall Test (Memory Test Postoperatively and Follow-up Study)

**Figure 6:**
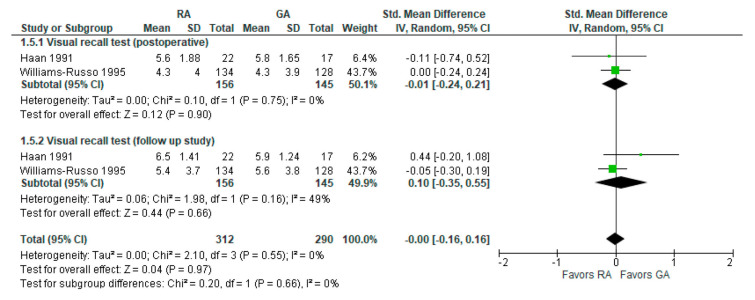
Memory tests (visual recall test) [19,26].

Visual recall test (memory test) results were reported in two [19,26] studies (Figure 6). In a visual recall test conducted four days postoperatively by Haan et al., 1991 [19] and one week postoperatively by Williams-Russo et al., 1995 [26], the model does not show any difference between RA and GA, and the result is insensitive to the exclusion of either study. The overall effect of the model on the results of the memory tests does not show any difference between RA and GA, and this result is insensitive to the exclusion of any study.

### 3.6. MMSE Score 24 h Postoperatively

**Figure 7:**
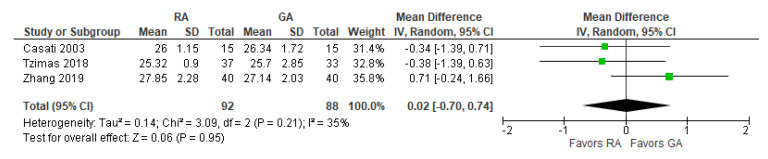
The MMSE score 24 h postoperatively [17,24,27].

The post-operative mini-mental state examination (MMSE) test score was reported in three [17,24,27] studies (Figure 7). The overall effect of the model does not show any difference between RA and GA, and this result is insensitive to the exclusion of any study. 

### 3.7. Reaction Time Three Months Postoperatively (ms)

**Figure 8:**
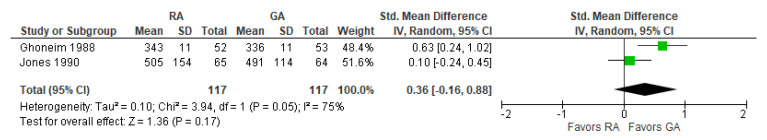
Post-operative reaction time three months postoperatively (ms) [18,20].

The post-operative reaction time was reported in two [18,20] studies (Figure 8). The overall effect of the model does not show any difference between RA and GA, but the result is sensitive to the exclusion of a study by Jones 1990 [20], in which case the model favors GA over RA.

### 3.8. Controlled Oral Word Association Test

**Figure 9:**
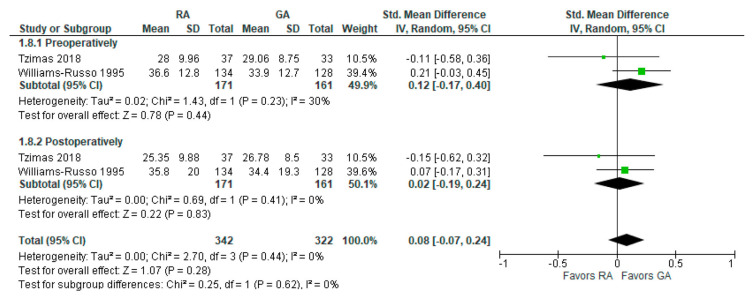
Controlled oral word association test [24,26].

The preoperative and post-operative controlled oral word association test results were reported in two [24,26] studies (Figure 9). The model shows no difference between RA and GA, and the result is insensitive to the exclusion of either study. We should note that Tzimas et al., 2018 [24] reported results of the test conducted 30 days postoperatively, and Williams-Russo et al., 1995 [26] one week postoperatively.

### 3.9. Digit Copying Test, PO Three Months

**Figure 10:**
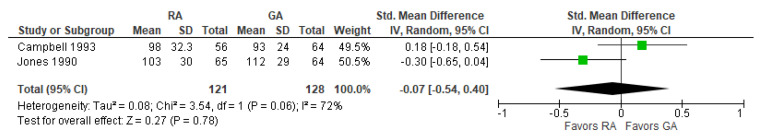
Digit copying test [16,20].

The results of the three-month post-operative digit copying test were reported in two [16,20] studies (Figure 10). The overall effect of the model does not show any difference between RA and GA, and the result is insensitive to the exclusion of either study. The model shows high heterogeneity (I^2^ = 72%).

### 3.10. Post-operative Death

**Figure 11:**
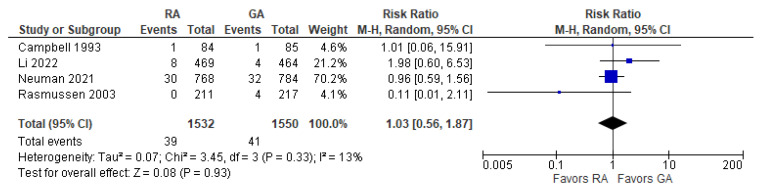
Incidence of post-operative death [10,16,21,22].

The overall effect of the model (Figure 11) does not favor the RA over the GA (the risk ratio with 95% CI: 1.03 [0.56, 1.87]). We should note that Campbell 1993 [16] reported deaths for the period of two to three months postoperatively (due to “probable myocardial infarction”), whereas Rasmussen 2003 [10] reported deaths within two days and three months (deaths due to “pulmonary embolism, heart failure, and unknown cause”). The causes of death vary from “probable myocardial infarction” after cataract surgery [16] to “pulmonary embolism, heart failure, and unknown cause” after joint replacement [10]. Li 2022 [21] and Neuman 2021 [22] reported 30-day and 60-day mortality, respectively. The model shows low heterogeneity (I^2^ = 17%).

### 3.11. Assessment of Methodological Quality (and Cochrane Risk of Bias Tool)

#### Quality Assessment

We report the Cochrane Risk of Bias in Table 1. Overall, seven studies were rated as low risk, four as some concerns, and two as high risk.

The GRADE summary of findings table of primary outcomes is reported in Table 2 and Supplementary File S2 “Addendum to the methodological assessment according to GRADE”. The certainty of evidence ranged from “high” to “low”. All the outcomes had a certain level of risk of bias as patient blinding was impossible due to the nature of the intervention.

## 4. Discussion

In this meta-analysis, we compared the differences in postoperative delirium and postoperative cognitive dysfunction between general and regional anesthesia. Although we tried to include the best quality and the most suitable studies, there were no statistically significant differences in any of the following outcomes and tests, including the incidence of POCD per-protocol analysis, the incidence of post-operative death, psychomotor/attention tests (preoperative/baseline, post-operative), memory tests (post-operative, follow up), MMSE score 24 h postoperatively, post-operative reaction time three months postoperatively, controlled oral word association test, digit copying test.

There were no differences in the incidence of POCD in general and regional anesthesia at one week postoperatively, three months, or total events (one week or three months). Furthermore, there was not even a single individual study that demonstrated a difference at any time in the post-operative period (early or late). Studies that evaluated the influence of general or regional anesthesia on the incidence of POD also did not show any difference between general or regional anesthesia.

It has been a constant question of what type of anesthesia is safer and less detrimental for the brain and other organs. Although it has been previously suggested that drugs used for general anesthesia could have neuroprotective effects, there is no strong evidence supporting this hypothesis [2]. Furthermore, previous preclinical studies have shown that anesthetic agents used for general anesthesia could induce neurotoxicity, and neuromodulation by general anesthetics could be harmful to cognition, especially in the developing and aging brain [28].

The fact that the current meta-analysis did not show any difference between regional and general anesthesia could be explained by the multifactorial nature of POCD and POD. Therefore, probably the type of anesthesia alone cannot affect the risk of POCD and POD.

Our systematic review has several limitations, including low statistical power, small sample size, heterogeneous group of patients with different diseases and comorbidities, and patients undergoing different surgical procedures. Another limitation is that different tests were used across the studies; therefore, it limited the possibility of comparing the outcomes of several studies within one analysis and creating the Forest plot. Some authors reported that surgeons and anesthesiologists did not follow the study protocol strictly. Finally, the protocols of general and regional anesthesia, including the drugs used for sedation, varied significantly in the included studies.

Cognitive dysfunction is identified using a battery of neuropsychiatric tests that offer a detailed assessment of higher cortical function rather than the usual neurological examination. It is difficult to determine the precise cause of POCD, for example, surgery-related or anesthesia-related, and these causes are currently almost inseparable. Therefore, both act as confounders of each other, obstructing an understanding of a causal relationship. Although the careful design of animal and human studies has been partially successful in separating the contributions of surgery and anesthesia to the development of POCD, there is still a high variability in the study design, creating limitations in the interpretation of these results.

It is generally challenging to study post-operative cognitive outcomes for several reasons. Patients may have preoperative, intraoperative, and post-operative risk factors that contribute to POCD and POD, although some patients may have multiple risk factors. Elderly patients undergoing surgical procedures generally have comorbidities, which are also considered risk factors for POD and POCD. Therefore, it is not always possible to equally randomize these patients without bias. The following predisposing factors have been linked to POD:

1. Preoperative factors: advanced age, history of alcoholism, cognitive impairment, neurocognitive disorders (e.g., psychosis, depression, dementia), hypertension, diabetes mellitus, chronic renal failure, anemia, chronic renal failure, electrolyte disorders, anemia, chronic steroid, and antipsychotic agents [29,30,31];
2. Intraoperative factors: duration of anesthesia and/or surgery, anesthetic drugs, intraoperative hypoxemia or/or hypotension, blood loss;
3. Post-operative risk factors: quality of sleep, the use of sedative agents, and post-operative pain intensity [32].

Interestingly, there was no difference between intravenous analgesia and neuraxial blocks for post-operative pain control in the developing POD; hence, the most important goal is to achieve a sufficient level of pain control, regardless of the analgesic option [33]. Therefore, regional anesthesia probably does not play a key role in reducing the risks of POD. Taking into consideration all the above-listed factors contributing to post-operative cognitive dysfunction, it is difficult to ascertain that these studies achieved unbiased results. The incidence of POCD in the included studies ranged from 10 to 20%. The authors reported a low recruitment rate because the majority of patients were unwilling to participate.

There are still many controversies about the POCD. Anecdotes about permanently diminished cognitive abilities after surgery might be compelling. It might also be assumed that the surgery and/or anesthesia can cause cognitive change. Nevertheless, the detection of cognitive decline after surgery is expected for several reasons. Thus, cognitive decline is common with aging, especially in patients with coexisting depression, diabetes mellitus, chronic obstructive pulmonary disease, and cardiovascular diseases. About half of people who are older than 60 years old undergo a surgical procedure. Consequently, it is likely that cognitive decline will initially be detected in some patients after surgery. Moreover, the preoperative cognitive function is not currently assessed. Many older patients already experience subclinical or undetected cognitive decline. It is also now recognized that rapid-onset dementia can develop over a period of weeks to months [34].

This meta-analysis does not favor regional anesthesia over general anesthesia in any of the studied neurocognitive outcomes; therefore, to date, we cannot recommend one method over another for reducing the hypothetical negative effect of general anesthesia on the brain. Taking into consideration the limitations of the previous studies and this meta-analysis, future RCTs with strict study protocols and enrollment of a homogeneous patient population might be more successful in determining the preferred method of anesthesia.

## 5. Conclusions

There was no statistically significant difference in the incidence of POCD per-protocol analysis, psychomotor/attention tests (preoperative/baseline, post-operative), memory tests (postoperatively, follow up), MMSE score 24 h postoperatively, post-operative reaction time three months postoperatively, controlled oral word association test, digit copying test. There were no differences in the incidence of POCD in general and regional anesthesia at one week postoperatively, three months, or total events (one week or three months). The incidence of post-operative mortality also did not differ between two groups.

Due to significant limitations of the available data and the current meta-analysis, we cannot make any conclusive recommendation regarding the use of the best anesthetic techniques to prevent post-operative delirium and post-operative cognitive discussion. The anesthetic technique should likely be personalized according to individual patient’s characteristics, health status, risk factors, types of surgery, and patient’s preferences.

## Figures and Tables

**Table 1:** Cochrane risk-of-bias tool.

Study	D1	D2	D3	D4	D5	Overall		
Zhang et al., 2019 [27]	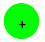	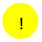	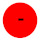	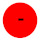	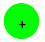	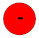	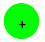	Low risk
Tzimas et al., 2018 [24]	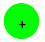	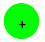	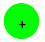	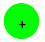	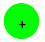	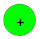	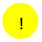	Some concerns
Silbert et al., 2014 [23]	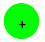	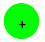	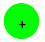	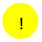	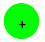	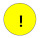	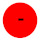	High risk
Rasmussen et al., 2003 [10]	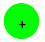	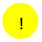	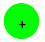	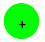	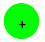	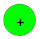	D1	Randomization process
Williams-Russo et al., 1995 [26]	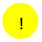	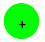	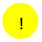	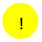	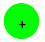	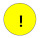	D2	Deviations from the intended interventions
Campbell et al., 1993 [16]	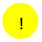	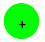	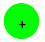	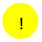	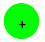	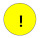	D3	Missing outcome data
Haan et al., 1991 [19]	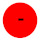	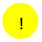	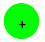	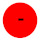	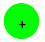	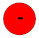	D4	Measurement of the outcome
Jones et al., 1990 [20]	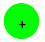	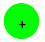	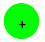	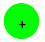	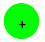	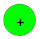	D5	Selection of the reported result
Ghoneim et al., 1988 [18]	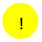	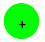	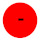	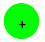	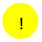	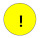		
Weber et al., 2009 [25]	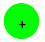	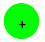	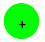	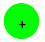	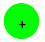	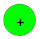		
Casati et al., 2003 [17]	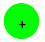	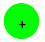	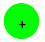	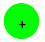	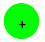	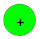		
Neuman et al., 2021 [22]	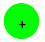	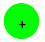	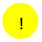	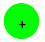	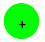	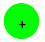		
Li et al., 2022 [21]	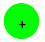	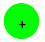	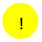	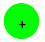	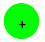	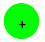		

Symbols: “+”—low bias, “-“—high bias, and “!”—unclear risk of bias.

**Table 2:** Summary of findings table of primary outcomes by GRADE. Patients: Patients undergoing any type of surgery; Intervention: Local anesthesia; Comparison: General anesthesia; Follow-up: In-hospital, one week, three months, and six months.

Outcomes	Standardized Mean Difference [95% CI]	Number of Patients (Studies)	Certainty of the Evidence (GRADE)
Incidence of PO delirium	1.10 [0.91, 1.33]	2963 (5)	⊕⊕⊕◯ Moderate ^a^
Incidence of POCD	1.14 [0.60, 2.13]	885 (2)	⊕⊕⊕◯ Moderate ^a^
Death	1.03 [0.56, 1.87]	3082 (4)	⊕⊕⊕⊕ High
Psychomotor/attention tests PO	0.05 [−0.06, 0.15]	1529 (6)	⊕⊕⊕⊕ High
Visual recall (PO and follow-up)	0.00 [−0.16, 0.16]	602 (2)	⊕⊕⊕⊕ High
Controlled oral word association test	0.08 [−0.07, 0.24]	664 (2)	⊕⊕⊕⊕ High
MMSE 24 h	0.02 [−0.70, 0.74]	180 (3)	⊕⊕◯◯ Low

^a^ due to the risk of bias and inconsistency. Abbreviations. CI, confidence interval; MMSE, mini-mental state examination; PO, post-operative; POCD, post-operative cognitive dysfunction. Symbols: ⊕⊕⊕⊕—“high certainty”, ⊕⊕⊕O—“moderate certainty” and ⊕⊕OO—“low certainty”.

## Data Availability

The data will be shared on request with the corresponding author.

## Supplementary Materials

The following supporting information can be downloaded at: https://www.mdpi.com/article/10.3390/jcm12103549/s1, Supplementary File S1. Characteristics of included studies. Supplementary File S2. Addendum to the methodological assessment according to GRADE.

## Registration

The protocol was registered in PROSPERO (registration number: CRD42022306582).
