# Supplementary material for: Postoperative Delirium and Cognitive Dysfunction after General and Regional Anesthesia: A Systematic Review and Meta-Analysis
Source: J Clin Med. 2023 May 18;12(10):3549. doi: 10.3390/jcm12103549 (PMC10219265; doi:10.3390/jcm12103549)
Supplement: Supplementary file 1 [file jcm-12-03549-s001.zip › jcm-2373222-supplementary.pdf]

Supplementary file

Author, citation	Country	Study design	Study goals	Age	N of patients: total (intervention / control)	Group	Surgery	General anesthesi a	ASA status	Local anesthetics, volume and concentration, adjuvants	Postoperative analgesia
Zhang, 2019	China	RCT	Primary – eye-opening time at 1, 3, 6, 24 hrs, MMSE, incidence of POCD at 24h, P300 wave	≥60 61-74 69.36 (2.57)	R, A: 80 (40/40)	SA: Spinal anesthesia GA: General anesthesia	Orthopedic surgery	Yes in GA group	NG	SA: L3-4 epidural anesthesia + 1 ml of 0.5% + 0.25% lidocaine if necessary GA: IV drip midazolam 0.05 mg/kg, propofol 1.5 mg/kg, fentanyl 0.4 µg/kg, and vecuronium bromide 0.1 mg/kg	NG
Tzimas, 2018	Greece	RCT	Primary – POCD at 30 days and possible differences between groups Secondary - delirium at 1 st, 2nd, 3rd and 4th postoperative day	>65 77.11 (6.5) 75.09 (6.08)	R: 72 (37/35) A: 68 (35/33)	SA: Spinal anesthesia GA: General anesthesia	Hip fracture surgery	Yes in GA group	I-III	SA: L3-L4 or L3-C5 spinal puncture (Fentanyl 20 mcg and ropivacaine 0.75% administered in volume according to the somatometric characteristics of the patient) GA: induction with fentanyl 3–5 µg.kg−1and propofol 1.5 mg.kg−1; maintained with Desflurane by adjusting end-tidal concentrations.	Postoperative analgesia to keep VAS<44 mm
Silbert, 2014	Australia	RCT	Primary - incidence of POCD at 3 months. Secondary - incidence of POCD at 7 days	≥55 66.9 (56-81) 63.9 (55-78) 68.1 (60-75)	SA/GA Rand: 98 (48/50) Treat: 92 (42/50) Anal at 7d: 87 (38/49) Anal at 3mon: 85(41/44) C: 26	SA: Spinal analgesia GA: General anesthesia C: Non-treatment control group	SA, GA: Extracorporeal shock wave lithotripsy (ESWL) to break kidney stones C: Hip osteoarthritis	Yes for GA group	NG	SA: T8 2 ml 0.5% heavy spinal bupivacaine, sedation avoided unless clinically indicated. GA: IV midazolam ≤0.05 mg kg−1, fentanyl ≤1 µg kg−1, and propofol 1–2 mg kg−1 C: Analgesics for hip osteoarthritis	POA: Non-opioids (paracetamol, celecoxib) unless clinically indicated
Aleksik, 2006	Germany		Primary - changes in cognitive function and affective state	68 (8) 62 (11)	33/25	LCA: Local cervical anesthesia LPA: Local peridural anesthesia	LCA: Carotid endarterectomy LPA: bypass revascularization for peripheral arterial occlusive	No	NG	LCA: Local cervical anesthesia LPA: Local peridural anesthesia	NG

Supplementary file

							disease				
Rasmussen, 2003	Denmark, data from 7 countries	RCT	Primary – Incidence of POCD	>60<br>71.1 (61.0-83.7)<br>70.8 (61.3-84.1)	R: 428 (211/217)<br><br>Completed 3 months: 340 (165/175)	RA: Regional anesthesia<br>GA: General anesthesia	Orthopedic, gynecological, urological, gastrointestinal, other surgeries	Yes in GA group	I-IV	RA: Spinal or epidural anaesthesia. Sedation with propofol permitted during regional anaesthesia at a level compatible with prompt arousal to a verbal stimulus.<br>GA: Normocapnia maintained. Neuraxial blockade or regional analgesia were not used.	RA: postoperative epidural analgesia
Williams-Russo, 1995	USA	RCT	Primary - memory, psychomotor, and language skills<br>Secondary – cardiovascular outcomes	Median 69	R: 262 (134/128)	EA: Epidural<br>GA: General anesthesia	Elective primary total knee replacement	Yes in GA group	NG	EA: L3-L4 or L2-L3 either lidocaine 2% or bupivacaine 0.75% + midazolam and fentanyl<br>GA: induction by thiopental sodium, fentanyl, vecuronium, maintained with fentanyl and inhaled N2O 70%	EA: >95% postoperative epidural anesthesia 12-72h<br>GA: IV analgesia 12-72h
Campbell, 1993	UK	RCT	Primary - differential changes in cognitive function<br>Secondary – influence of episodes of hypotension or hypoxia on the outcome	>65<br>77.3 (7.71)<br>77.9 (7.22)	R: 169 (84/85)<br>A (pre, 24h, 2 weeks): 157 (80/77)<br>A (3 months): 120 (56/64)	LA: Local anesthesia<br>GA: General anesthesia	Elective cataract extraction	Yes in GA group	NG	LA:<br>Retrobulbar or peribulbar blockade using lignocaine (2%) with or without bupivacaine (0.5%) + topical amethocaine (1 %). Facial nerve block for most patients.<br>GA: induction by thiopentone (34<br>mg.kg-1) or methohexitone (1 mg.kg-1) and vecuronium (0.1 mg.kg-1). Fentanyl (0.05 mg) if required.	Paracetamol (1 g)
Haan, 1991	Netherlands	Prospective partially randomized	Primary - effect on cognitive functions and whether MMSE can be used as a tool to measure and predict a postoperative decrease of	Mean 71.8 (6.0), range 63.3 – 86.5	R + Non-R: 37 (22/15) + 12 (5/7)<br>Total 49 (27/22)	S: Spinal anesthesia<br>GA: General anesthesia	Elective transurethral prostatectomy for benign prostatic hyperplasia	Yes in GA group	I-III	Sedation for all patients (temazepam 10 mg to 20 mg (n = 45) or lorazepam 1 mg to 2 mg (n = 8)).<br>SA: 1% lidocaine + T8, T10 hyperbaric bupivacaine 0.5% (2 mL to 3 mL in 8% dextrose)<br>GA: induction by thiopentone (3 to 4 mg/kg body weight) or	Symoron (1 mg/10 kg body weight) every 6 hours<br>during 24 hours post-operatively.

			cognitive function							etomidate (0.3 to 0.4 mg/kg body weight), maintained with nitrous oxide-oxygen mixture (fiO2: 0.33), fentanyl (0.1 mg) for added analgesia, pancuronium, and enflurane (0.6%)	
Jones, 1990	UK	RCT	Primary - scores achieved in tests of cognitive function and functional competence	≥60	R: 146 (72/74) + 50 Non-R controls T: 146 (63/85) A: 129 (65/64)	RA: Regional anesthesia GA: General anesthesia	Elective hip or knee replacement.	Yes in GA group	NG	RA: IV midazolam (1 mg increments up to 10 mg). L2-3 or L3-4 2-3 ml of 0 5% bupivacaine in 8% dextrose. GA: induction by thiopentone, followed by pancuronium. Oxygen, nitrous oxide, and halothane supplemented by fentanyl (up to 0.2 mg).	IM morphine, 10 mg every four hours as required.
Ghoneim, 1988	USA	RCT	Primary - within-subject change in perceived and actual mental abilities	61.9 (1.8) 60.1 (2.3)	R, A: 105 (52/53) Of 52: 38 subarachnoid, 14 epidural	RA: Regional anesthesia: subarachnoid or epidural block GA: General anesthesia	Total knee arthroplasty, total knee arthroplasty, vaginal hysterectomy, transurethral prostatectomy	Yes in GA group	2.16 (0.1) 2 (0.09)	Subarachnoid: L3-L4 tetracaine (Pontocaine) in a hyperbaric dextrose 5%. Epidural: lumbar anesthesia 0.5% bypivacaine (Marcaine) with 1:200000 epinephrine. GA: induction with thiopental, maintaine with nitrous oxide in oxygen and isoflurane (65%), enflurane (26%) or halothane (9%). Fentanyl and muscle relaxants occasionally administered.	According to usual routine

S1. Characteristics of included studies.
RCT-randomized controlled trial;
RA- regional anesthesia;
GA-general anesthesia;
VAS-visual analog scale;
NG-not given;

Supplementary file

	Incidence of postoperative delirium	POCD per-protocol analysis at 1 week	POCD per-protocol analysis at 3 months	POCD per-protocol analysis at 1 week and 3 months	Digital span test preoperatively	Digital symbol test preoperatively	Trail making test A preoperatively	Trail making test B preoperatively	Psychomotor/attention tests preoperatively
**Risk of bias**	**Some**	**Low**	**Low**	**Low**	**Some**	**Some**	**Some**	**Yes**	**Yes**
Lack of allocation concealment	No	No	No	No	No	No	No	No for most	No for most
Lack of blinding	Yes for most	Yes	Yes	Yes	Yes	Yes	Unclear	Yes	Yes
Incomplete accounting of patients and outcome events	Yes	No	No	No	Yes	Yes	Yes	Yes	Yes
Selective outcome reporting	No	No	No	No	No	No	No	No	No

Other limitations	No	No	No	No	No	No	No	No	No
**Inconsistency**	**Some**	**Yes**	**Yes**	**Some**	**Yes**	**No**	**No**	**No**	**Some**
I^2 (unexplained heterogeneity of results)	No	Yes	Yes	Yes	Yes	No	No	No	Yes
Wide variance of point estimates	Yes	Yes	Yes	No	Yes	No	No	No	No
Confidence intervals (CIs) do not overlap	No	No	No	No	Yes	No	No	No	No
**Indirectness**	**No**	**No**	**No**	**No**	**No**	**No**	**No**	**No**	**No**
Differences in population	No	No	No	No	No	No	No	No	No
Differences in interventions	No	No	No	No	No	No	No	No	No
Differences in outcome measures	No	No	No	No	No	No	No	No	No

Indirect comparisons	No	No	No	No	No	No	No	No	No
**Imprecision**	**No**	**Yes**	**Yes**	**Some**	**Yes**	**No**	**No**	**No**	**No**
Few patients	No	Yes	Yes	No	No	No	No	No	No
Wide confidence interval (CI)	No	Yes	Yes	Yes	Yes	No	No	No	No
**Positive**	**No**	**No**	**No**	**No**	**Yes**	**Yes**	**Yes**	**Yes**	**Yes**
RR>2 or RR<0.5<br>RR>5 or RR<0.2	No	No	No	No	Yes	Yes	Yes	Yes	Yes
Dose-response gradient	No	No	No	No	No	No	No	No	No
Effect of plausible residual confounding	No	No	No	No	No	No	No	No	No

Supplementary file

	Digit span test postoperatively	Digital symbol test postoperatively	Trail making test A postoperatively	Trail making test B preoperatively	Psychomotor/attention tests postoperatively	Verbal recognition test postoperatively	Visual recall test postoperatively	Memory tests postoperatively	Verbal recognition test follow-up
**Risk of bias**	**Some**	**Some**	**Some**	**Yes**	**Yes**	**Yes**	**Some**	**Yes**	**Yes**
Lack of allocation concealment	No	No	No	No for most	No for most	No for most	No	No for most	No for most
Lack of blinding	Yes	Yes	Unclear	Yes	Yes	Yes	Yes	Yes	Yes
Incomplete accounting of patients and outcome events	Yes	Yes	Yes	Yes	Yes	Yes	Yes	Yes	Yes
Selective outcome reporting	No	No	No	No	No	No	No	No	No
Other limitations	No	No	No	No	No	No	No	No	No

Supplementary file

**Inconsistency**	**No**	**No**	**Some**	**Some**	**No**	**Some**	**No**	**No**	**No**
I^2 (unexplained heterogeneity of results)	No	No	Yes	Yes	No	Yes	No	No	No
Wide variance of point estimates	No	No	No	No	No	No	No	No	No
Confidence intervals (CIs) do not overlap	No	No	No	No	No	No	No	No	No
**Indirectness**	**No**	**No**	**No**	**No**	**No**	**No**	**No**	**No**	**No**
Differences in population	No	No	No	No	No	No	No	No	No
Differences in interventions	No	No	No	No	No	No	No	No	No
Differences in outcome measures	No	No	No	No	No	No	No	No	No
Indirect comparisons	No	No	No	No	No	No	No	No	No

Supplementary file

**Imprecision**	**No**	**No**	**No**	**No**	**No**	**Some**	**No**	**No**	**No**
Few patients	No	No	No	No	No	No	No	No	No
Wide confidence interval (CI)	No	No	No	No	No	Yes	No	No	No
**Positive**	**Yes**	**Yes**	**Yes**	**Yes**	**Yes**	**Yes**	**Yes**	**Yes**	**Yes**
RR>2 or RR<0.5<br>RR>5 or RR<0.2	Yes	Yes	Yes	Yes	Yes	Yes	Yes	Yes	Yes
Dose-response gradient	No	No	No	No	No	No	No	No	No
Effect of plausible residual confounding	No	No	No	No	No	No	No	No	No

Supplementary file

	Visual recall test follow-up	Memory tests follow-up	MMSE score 24 h postoperatively	Reaction time 3 months postoperatively (ms)	Controlled oral word association test preoperatively	Controlled oral word association test postoperatively	Controlled oral word association test preoperatively and postoperatively	4.0 digit copying test	Postoperative death
**Risk of bias**	**Some**	**Yes**	**No**	**Some**	**Some**	**Some**	**Some**	**Yes**	**Yes**
Lack of allocation concealment	No	No for most	No	No	No	No	No	Some	Unclear for 1 study
Lack of blinding	Yes	Yes	Unclear	No	Unclear	Unclear	Unclear	Some	Yes
Incomplete accounting of patients and outcome events	Yes	Yes	No	Yes	Yes	Yes	Yes	Yes	Yes
Selective outcome reporting	No	No	No	No	No	No	No	No	No

Other limitations	No	No	No	No	No	No	No	No	No
**Inconsistency**	**No**	**No**	**Yes**	**Some**	**Some**	**No**	**No**	**Yes**	**Some**
I^2 (unexplained heterogeneity of results)	No	No	Yes	Yes	Some	No	No	Yes	Low
Wide variance of point estimates	No	No	Yes	No	No	No	No	Yes	Yes
Confidence intervals (CIs) do not overlap	No	No	No	No	No	No	No	No	No
**Indirectness**	**No**	**No**	**No**	**No**	**No**	**No**	**No**	**No**	**No**
Differences in population	No	No	No	No	No	No	No	No	No
Differences in interventions	No	No	No	No	No	No	No	No	No
Differences in outcome measures	No	No	No	No	No	No	No	No	No

Indirect comparisons	No	No	No	No	No	No	No	No	No
**Imprecision**	**No**	**No**	**Yes**	**No**	**No**	**No**	**No**	**Some**	**Some**
Few patients	No	No	Yes	No	No	No	No	No	No
Wide confidence interval (CI)	No	No	Yes	No	No	No	No	Yes	Yes
**Positive**	**Yes**	**Yes**	**Yes**	**Yes**	**Yes**	**Yes**	**Yes**	**Yes**	**Yes**
RR>2 or RR<0.5<br>RR>5 or RR<0.2	Yes	Yes	Yes	Yes	Yes	Yes	Yes	Yes	Yes
Dose-response gradient	No	No	No	No	No	No	No	No	No
Effect of plausible residual confounding	No	No	No	No	No	No	No	No	No

S2. Addendum to the methodological assessment by GRADE.

Patients: Patients undergoing any types of surgery; Intervention: Local anesthesia; Comparison: General anesthesia; Follow-up: In-hospital, 1 week, 3 months, 6 months

Supplementary file
